# Salivary Cortisol Levels and Depressive Symptomatology in Consumers and Nonconsumers of Self-Help Books: A Pilot Study

**DOI:** 10.1155/2016/3136743

**Published:** 2015-12-29

**Authors:** Catherine Raymond, Marie-France Marin, Anne Hand, Shireen Sindi, Robert-Paul Juster, Sonia J. Lupien

**Affiliations:** ^1^Centre for Studies on Human Stress, Research Centre, Institut Universitaire en Santé Mentale de Montréal, Montreal, Canada H1N 3M5; ^2^Department of Neuroscience, University of Montreal, Montreal, Canada H3C 3J7; ^3^Integrated Program in Neuroscience, McGill University, Montreal, Canada H3A 3R1; ^4^Department of Psychiatry, Faculty of Medicine, University of Montreal, Montreal, Canada H3T 1J4

## Abstract

The self-help industry generates billions of dollars yearly in North America. Despite the popularity of this movement, there has been surprisingly little research assessing the characteristics of self-help books consumers, and whether this consumption is associated with physiological and/or psychological markers of stress. The goal of this pilot study was to perform the first psychoneuroendocrine analysis of consumers of self-help books in comparison to nonconsumers. We tested diurnal and reactive salivary cortisol levels, personality, and depressive symptoms in 32 consumers and nonconsumers of self-help books. In an explorative secondary analysis, we also split consumers of self-help books as a function of their preference for problem-focused *versus* growth-oriented self-help books. The results showed that while consumers of growth-oriented self-help books presented increased cortisol reactivity to a psychosocial stressor compared to other groups, consumers of problem-focused self-help books presented higher depressive symptomatology. The results of this pilot study show that consumers with preference for either problem-focused or growth-oriented self-help books present different physiological and psychological markers of stress when compared to nonconsumers of self-help books. This preliminary study underlines the need for additional research on this issue in order to determine the impact the self-help book industry may have on consumers' stress.

## 1. Introduction

The World Health Organization predicts that by the year 2020, depression will be the first cause of invalidity in the world followed by cardiovascular disease [1]. Although various psychological and pharmacological treatments exist for the treatment of depression (for a review, see [2]), difficult access to psychotherapy due to monetary or transportation issues and/or low acceptance of antidepressant treatments has led to the development of other forms of treatments [3]. In recent years, there has been a rise in the use of self-help treatments that provide users with information on how to self-identify their problems and propose methods to overcome them [3].

Self-help exists in a variety of mediums. The most frequent form of delivery includes books (bibliotherapy) and use of the internet (internet-based therapy; for a review, see [3, 4]). Moreover, self-help treatment can be guided or unguided. Guided self-help treatment implies that some form of support from a therapist is delivered to the patient, either through self-help booklets developed by health professionals or scientists, or via support provided directly by a therapist in addition to utilization of the self-help material [5]. In contrast, unguided self-help represents the use of “self-help books” available in bookstores with no additional support from a health professional [4, 6]. Unguided self-help books represent books written by recognized or unrecognized specialists in the field that provides guidance on how to live a better life, be happy, and so forth [4, 7].

Two dimensions of self-help are generally proposed in unguided self-help books [4, 6], that is, problem-focused or growth-oriented [4]. Problem-focused self-help books represent books that extensively discuss the nature of problems one can encounter and how to recognize and circumvent them [4] (this category of self-help books has also been named “*victimization* books” [6]). In contrast, growth-oriented books present inspirational messages about life and happiness and propose various methods of coping and development of new skills [4] (this category of self-help books has also been named “*empowerment* books” [6]).

Meta-analyses have shown that guided self-help interventions for depression are more effective than absence of treatment, and guided self-help interventions present similar efficacy to psychotherapies and/or antidepressants [2, 8, 9]. Moreover, guided self-help interventions are now recommended by the National Institute for Health and Clinical Excellence [10]. Although guided self-help interventions presented in books or via the internet have been extensively studied [2, 11, 12], unguided self-help books have received very little attention. Some studies suggest that reading problem-focused self-help books can have positive effects in the treatment of some problematics such as marital conflict [13] and general emotional disorders [14], and others suggest that unguided self-help books could be used to prevent the incidence of depression in high risk groups [15]. However, at this point, there is a lot of cynicism about the potentially positive effects of unguided self-help books, with some authors claiming that self-help books are fraudulent [16], and others suggesting that buying self-help books may be part of a “false hope syndrome” [17]. For many authors, the major limitation of unguided self-help books is their “one-size fits all” approach in which advice is given without taking into account the personality and/or diagnosis and/or personal circumstances of the reader [16–18].

This later point brings attention to the lack of information that exists on the type of readership of unguided self-help books. The few studies that were performed to date showed that consumers of self-help books come from all levels of educational backgrounds, socioeconomic status, and positions, although women tend to consume more self-help books than men [13]. Notwithstanding, the literature is inconsistent in describing whether consumers of self-help books differ from nonconsumers in terms of personality [19]. One study showed that consumers of self-help books present higher neuroticism than nonconsumers [20], a second study did not find such a difference [4, 21], and a third reported that reading self-help books is associated with an increase in self-actualization [13].

Although these data are interesting, they do not inform us about the characteristics of self-help book readership. Indeed, studies assessing why certain people are attracted to self-help books propose that many adults are active consumers of self-help books as a way of self-diagnosing and/or “treating” their own psychological distress, and that this would mainly result from the stigma surrounding depression in adults [22, 23]. In this sense, the active proliferation of the self-help book industry would mainly reflect the underlying depressive symptomatology of individuals, and this industry would be highly successful because individuals need some sort of autotreatment to alleviate their depressive mood and/or disorder. If this is the case, one could predict that active consumers of self-help books might present increased stress physiology and increased depressive symptomatology when compared to nonconsumers of self-help books. The goal of this study was to test this hypothesis.

Impairment in the regulation of the hypothalamic-pituitary-adrenal (HPA) system has been reported in acute and/or chronic episodes of depression [24, 25]. The impaired negative feedback of the HPA system ultimately leads to hypersecretion of CRF, shifting the activity of the HPA axis to greater production of glucocorticoids (cortisol in humans; for a review, see [26]). In this first pilot study, we assessed whether consumers of unguided self-help books present differences in diurnal levels of cortisol, stress reactive cortisol levels, depressive symptoms, and personality traits in comparison to nonconsumers. Personality and depressive symptomatology are important factors to measure in consumers of self-help books as they could potentially be important predictors of increased stress reactivity and/or depressive symptomatology. In line with the goals of pilot studies (for a review on pilot studies, see [27]), we performed this first small scale preliminary study in order to evaluate the feasibility of studying self-help book consumers and potential adverse events related to these types of studies. Most importantly, to guide future research, we aimed to generate effect sizes for our dependent variables (cortisol levels, depressive symptomatology, and personality factors) in order to determine the appropriate sample size needed for a larger experimental study on this issue that has received no empirical evidence.

## 2. Materials and Methods

### 2.1. Recruitment and Group Classification

The definition of “self-help books” that we used in this research project is the definition given by the neuropsychologist Paul Pearsall who defines self-help books as “*Books that give advice on how to change your life, attain happiness, find true love, lose weight, and more*” [7]. We defined “consumers of self-help books” as individuals who have bought or browsed a minimum of four self-help books in the previous year. We felt that including only individuals who have bought (and not “browsed”) four self-help books might bias the sample toward people from higher socioeconomic status, which could then have a significant impact on the results. Questions about the number and types of books bought and/or browsed by the participants were asked during a recruitment phone interview. Participants defined as “consumers of self-help books” were asked to provide the names of these books during the phone interview in order to ascertain whether they fell into our category of consumers of self-help books.

Online recruitment was performed using advertisements posted on general or university websites. Since the purpose of the study was to compare two different populations (self-help books' consumers and nonconsumers), two different types of advertisement were used. Nonconsumers were recruited via an advertisement featuring a study on personality traits and stress, without any mention on self-help books consumption. This procedure was used to ensure that the nonconsumer group was not composed of people “against” this type of literature but only “not attracted” to it. These potential nonconsumer participants were then screened on the phone and additional questions were asked to validate that they had never read or browsed that kind of self-help literature and that they were not attracted to it. Only those individuals who did not read self-help books and were not attracted to them were retained in the nonconsumer group.

Self-help book consumers were recruited via an advertisement stating that we were looking for adults who were active consumers of self-help books for a study on personality and stress. During their visit to the lab, participants from that group were evaluated on their preference for problem-focused* versus* growth-oriented self-help books using a classification task that we developed. In this task, we presented the consumer group with 10 books and, after giving them 10 minutes to browse the various books, we asked them to sort out the five books that they would buy given the opportunity. Five of the ten books proposed a growth-oriented approach (e.g., “*The Power of Positive Thinking*”), while five of them proposed a problem-focused approach (e.g., “*How Can I Forgive You?: The Courage to Forgive, the Freedom Not To*”). The 5 books in each category are presented as follows.

*Books Used to Assess Preference for Growth-Oriented (Books #1 to #5) versus Problem-Focused (Books #6 to Book #10) Self-Help Books.* Growth-Oriented Self-Help Books are the following:

1. “*The Power of Positive Thinking*” by Norman Vincent Peale, 1952.
2. “*How to Stop Worrying and Start Living*” by Dale Carnegie, 1990.
3. “*You're Stronger than You Think*” by Peter Ubel, 2006.
4. “*You Can Be Happy No Matter What*” by Richard Carlson, 2006.
5. “*Choices That Change Lives: 15 Ways to Find More Purpose, Meaning, and Joy*” by Hal Urban, 2006.

Problem-Focused Self-Help Books are as follows:

(6) “*Why Is It Always About You*?:* Saving Yourself from the Narcissists in Your Life*” by Sandy Hotchkiss, 2003.
(7) “*I'm Ok, You're My Parents*” by Dale Atkins, 2004.
(8) “*Shame and Guilt*” by Jane Middelton-Moz, 1990.
(9) “*Self Nurture: Learning to Care for Yourself As Effectively As You Care for Everyone Else*” by Alice D. Domar and Henry Dreher, 2001.
(10) “*How Can I Forgive You?: The Courage to Forgive, the Freedom Not To*” by Janis A. Spring, 2005.

A ratio of growth-oriented/problem-focused preference was calculated by adding the number of books from each pole that fell within the category of “books to buy” by the participants. For example, if a participant stated that they would buy three growth-oriented books and two problem-focused books, this participant received a ratio of 3/2 = 1.5. With this ratio, the larger the number, the greater the attraction to growth-oriented books and vice versa for problem-focused books. Participants displaying a ratio of 4 and above were classified in the growth-oriented group as scores lower than 4 were closer to chance level for preference assessment. When presented with books, participants were not aware that the goal of this task was to determine their attractiveness to growth-oriented* versus* problem-focused books. The reason for this is that it can be predicted that most people would choose not to select problem-focused books if told about the two poles (growth-oriented* versus* problem-focused), given the negative social value that may be attached to problem-focused self-help books.

### 2.2. Participants

Participants from both groups were screened over the phone prior to recruitment in order to make sure that they fulfilled our inclusion criteria. Exclusionary criteria included presence or history of neurological or psychiatric conditions, diabetes, respiratory disease, asthma, infectious illness, thyroid or adrenal dysfunctions, obesity (body massive index > 30), any glucocorticoid or cardiovascular altering medications (e.g., antidepressants, diuretics, antiasthmatics, and b-blockers), and excessive use of drugs or alcohol. Smoking was an exclusion criterion due to its known effect on HPA axis regulation [19].

Thirty-two healthy men and women aged between 18 and 65 (*M* = 36.03 ± 16.09) participated in this study. Eighteen self-help consumers (75% female) and 14 nonconsumers (75% female) were recruited. The average age of the consumers was 38.33 years old (±3.5) and 33.07 years old (±4.72) for the nonconsumers. Within the group of self-help books consumers, 11 individuals were classified as having a preference for problem-focused books (hereon referred to as the “problem-focused group”) and 7 were classified as having a preference for growth-oriented books (hereon referred to as the “growth-oriented group”). Three women were menopausal (one in each condition) and all others were tested in the follicular phase of their menstrual cycle. Women on hormonal therapy were not included in this study. All participants provided written informed consent and were compensated for their participation in the study.

This study was approved by the Research Ethics Board of the Mental Health University Institute respecting the Canadian Tri-Council's Policy statement for the ethical conduct for experimentation using humans, guided by the World Medical Association's Declaration of Helsinki.

### 2.3. Questionnaires

#### 2.3.1. Personality Traits

We measured personality traits in order to determine whether preference for problem-focused or growth-oriented books would be associated with personality traits that could predict cortisol levels. Personality traits were measured using the NEO Five Factor Inventory (NEO-FFI). This 60-item personality inventory was developed as a short form of the NEO-PI [28]. The subscales include “neuroticism,” “extraversion,” “openness to new experiences,” “agreeableness,” and “conscientiousness”. Participants are asked to respond on a Likert-scale with the extent to which they agree with each item (“strongly agree” to “strongly disagree”). The mean coefficient alpha for the revised inventory scale was 0.77.

#### 2.3.2. Locus of Control

Since low sense of control is linked to the cortisol stress response [29], locus of control was measured in order to explain any potential physiological stress response differences between groups. We administered the Belief in Competence and Control Questionnaire (BCC). Using a six-point Likert scale (“not at all true” to “very true”), the BCC yields four scales including “self-concept of own competence,” “control expectancy: internality,” “control expectancy: externality,” and “control expectancy: chance control” [30]. The mean alpha for this questionnaire is 0.82 for young students and 0.83 for the elderly.

#### 2.3.3. Self-Esteem

Self-esteem was measured using the 10-item Rosenberg Self-Esteem Scale (RES [31]), which is a unidimensional scale that measures personal worth, self-confidence, self-respect, and self-depreciation. Participants are asked to respond on a four-point scale with the degree to which they agree with each item (“strongly agree” to “strongly disagree”). The scale shows good reliability (*α* = 0.80) and is a valid test of global self-worth.

#### 2.3.4. Depressive Symptomatology

Self-reported depressive symptoms were assessed using the 21-item Beck Depression Inventory II (BDI II [32]), which is a unidimensional scale that assesses diverse psychological and physiological symptoms related to depression on a four-point scale. The BDI's total score ranges from 0 to 63, displaying a continuum of depression related symptoms. The scale has been found to show good reliability (0.92). Total sum scores were used in the present analysis.

### 2.4. Diurnal Cortisol Secretion

All participants were provided with a saliva kit to bring home. They were asked to provide samples on two different days, separated by 3 days, with the first day of sampling starting 3 days after their visit to our laboratory. Saliva was collected using passive drool at the time of awakening and 30 minutes after awakening in order to calculate the “Cortisol Awakening Response” (CAR [33]). It has been reported that during the first hour after awakening, cortisol levels show an acute increase [33]. Cortisol determination during this time of day appears to represent a response of the HPA axis to an endogenous stimulation and is a reliable indicator of diurnal HPA activity [34]. Participants were also asked to provide three additional samples at 14:00 and 16:00 and before bedtime. These sampling times have been shown in previous studies to be reliable markers of the diurnal cycle of cortisol secretion [35, 36]. As the nonadherence to saliva sampling in ambulatory settings has been shown to exert a significant impact on the resulting cortisol profile [37], a “daily sampling questionnaire” was also completed. Individuals were asked to record the exact time of each saliva sample to assess participants' compliance.

### 2.5. Stress Reactivity

Participants were exposed to the Trier Social Stress Test (TSST [38]). The TSST is an established and highly effective psychosocial stress paradigm used to provoke activation of the HPA axis. The version of the TSST that we used in the current study was somewhat different from the original version as we used a “Panel-out” (judges behind a false mirror) instead of a “Panel-in” (judges in the same room as the participants) condition. The reason why we made the decision to use the Panel-out condition in the current study is that the research assistants who acted as judges in our experiment were younger than the participants. We have shown in previous studies that environmental factors such as age of research assistants can lead to a spurious stress response in some individuals [39, 40], and, consequently, we wanted to limit contact between our participants and the judges. The Panel-out version of the TSST was used in many of our studies. While one study reported no significant differences between the Panel-in and the Panel-out conditions in men [41], another study has reported higher cortisol reactivity in the Panel-in compared to the Panel-out condition in women [42]. Recently, our laboratory found no significant differences in terms of cortisol reactivity between the Panel-in and Panel-out condition when comparing 140 men, women in the luteal phase of their menstrual cycle, and women taking oral contraceptives. Therefore both conditions induce a stress response (article in preparation).

In summary, the TSST involves an anticipation phase (10 minutes) and a test phase that comprises 10 minutes of public speaking. The test phase is divided into a mock job interview (5 minutes) followed by mental arithmetic (5 minutes). Throughout their performance, participants face a one-way mirror and a camera. Behind this mirror, two confederates act as judges and pretend to be experts in behavioral analysis while observing the participants and communicating with them via an intercommunication system. Participants underwent the TSST in the afternoon between 13:30 and 16:30. A total of eight saliva samples for cortisol determination were obtained at −20 min and −10 min (baseline), immediately before the TSST as well as +10, +20, +30, +40, and +50 min after the TSST began.

### 2.6. Procedure

During recruitment, potential participants were told on the phone that the study consisted of one testing day, lasting two hours, and two days of saliva sampling at home were required following the testing session. All participants were tested at the Douglas Institute Research Center.

For the laboratory visit, participants were tested in the afternoon in order to obtain adequate cortisol reactivity to the psychosocial stressor and to control for possible differential effects of the circadian cortisol patterns. Upon arrival at the laboratory, participants were asked to read and sign an informed consent form. Thereafter, they were asked to answer the psychological questionnaires, which took approximately 15 minutes. Participants provided saliva samples by filling a small plastic vial with 1 mL of pure saliva (i.e., passive drool). Participants were instructed about the TSST and prepared their mock job interview speech during a 10-minute anticipation phase. Participants then had to do the verbal (5 minutes) and mental arithmetic (5 minutes) tasks. After the recovery period, they were debriefed with regard to the goal of the public speaking task. Participants were debriefed about the general hypothesis of the study when they brought the home saliva kit back to the lab.

### 2.7. Salivary Cortisol Assays

Salivary samples were maintained at –20°C until time of cortisol concentration determination. Salivary cortisol concentrations were determined in Dr. Dominique Walker's laboratory at the Douglas Institute Research Center by radioimmunoassay using a kit from DSL (Diagnostic System Laboratories, Inc., Texas, USA). Total binding and nonspecific binding typically range between 47–63% and 0.5–1.5%, respectively. The intra-assay and interassay coefficient of variation for these studies are 4.6% and 5%, respectively. The limit of detection of the assay is 0.01 dl, and all samples were assayed in duplicates.

### 2.8. Statistical Analysis

All the analyses were done in two separate sets. The first set of analyses was done with Group (2 levels: consumer* versus* nonconsumer) as the independent variable to test whether as a group, consumers of self-help books present different psychoneuroendocrine profiles when compared to nonconsumers of self-help books. In the second set of analyses, consumers of self-help books were split as a function of their preference for growth-oriented or problem-focused books and compared with the nonconsumer group, using Group (3 levels: growth-oriented, problem-focused, and nonconsumer) as the independent variable.

For each analysis, personality traits (as measured by the five NEO subscales “neuroticism,” “extroversion,” “openness,” “agreeableness,” and “conscientiousness”), locus of control, self-esteem, and depressive symptoms were included in univariate ANOVAs. For cortisol, both diurnal and reactive cortisol values followed a normal distribution and, for this reason, raw data of cortisol were used for all analyses. For each salivary cortisol analysis, sex and body mass index (BMI) were entered as covariates as these are factors associated with cortisol production [43]. Greenhouse-Geisser values were used when the assumption of sphericity was violated. Diurnal cortisol secretion was calculated using the mean concentration of cortisol for each sample on both days of saliva sampling, resulting in five cortisol means. In order to determine whether self-help book use was related to diurnal cortisol secretion, we calculated the CAR as well as using the trapezoidal method to calculate area under the curve with respect to ground (AUCg; basal cortisol). In order to determine whether self-help book use was related to reactive cortisol secretion, we calculated the area under the curve relative to increase (AUCi; reactive cortisol) [44]. These analyses were made in order to determine whether there were significant group differences in terms of basal and reactive cortisol levels between groups. To ascertain the participant's compliance regarding the diurnal saliva sampling, time when saliva samples were taken was computed into a mean in each group and ANOVAs were used to calculate whether there were significant group differences.

Finally, we calculated the effects size for the comparison between consumers* versus* nonconsumers and the comparison between preference for growth-oriented or problem-focused books in order to determine (1) the statistical power of the significant differences observed and (2) the appropriate sample size for a larger full scale study.

## 3. Results

### 3.1. Assessment of Feasibility and Adverse Events from This Pilot Study

In terms of feasibility, we found it quite easy to recruit consumers of self-help books as no differences were observed in terms of time and cost of recruitment of this population compared to other populations we have tested in the past. Recruitment of nonconsumers was more time consuming because we had to validate* a posteriori* the nonconsumption of self-help books in the individuals calling us to participate in the research but, overall, the burden was not high on recruitment. No adverse events were reported during recruitment and testing, although the research assistants working on this project reported that the testing of consumers of self-help books took generally longer than testing of nonconsumers because consumers were generally more verbal and interacted more with the assistants during testing.

### 3.2. Preliminary Analyses

**Figure 1:**
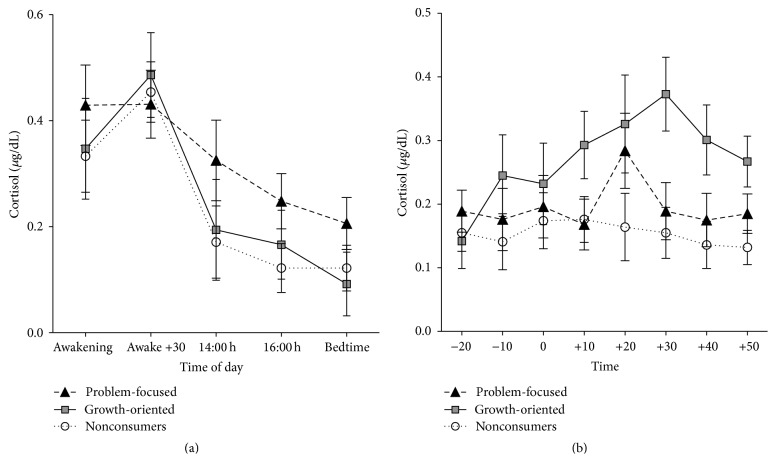
Repeated-measures of (a) diurnal cortisol and (b) reactive cortisol as a functioning of groups based on preference for problem-focused or growth-oriented self-help books. These graphs are used strictly to represent the mean (standard error bars) cortisol concentrations and to show the magnitude of the cortisol response to the TSST in each of the groups tested. As such, they have no relation to the statistical model employed that otherwise used the composite measure of area under the curve for cortisol levels (basal, reactive, and CAR).

Figure 1 shows that participants displayed a normal diurnal cortisol rhythm as well as an increase in cortisol in response to the TSST. Preliminary analysis also revealed that groups did not differ in terms of time of saliva sampling (all *P* values > 0.763) and that groups did not differ in terms of age, BMI, years of education, or sex of the participants (all *P* values > 0.165). Also, no group differences were observed for personality traits (all *P* values > 0.112), locus of control (all *P* values > 0.162), and self-esteem (all *P* values > 0.295) when we contrasted the consumers to the nonconsumers, and when we split the consumers into those individuals with a preference for growth-oriented or problem-focused books.

### 3.3. Consumers* versus* Nonconsumers of Self-Help Books

**Figure 2:**
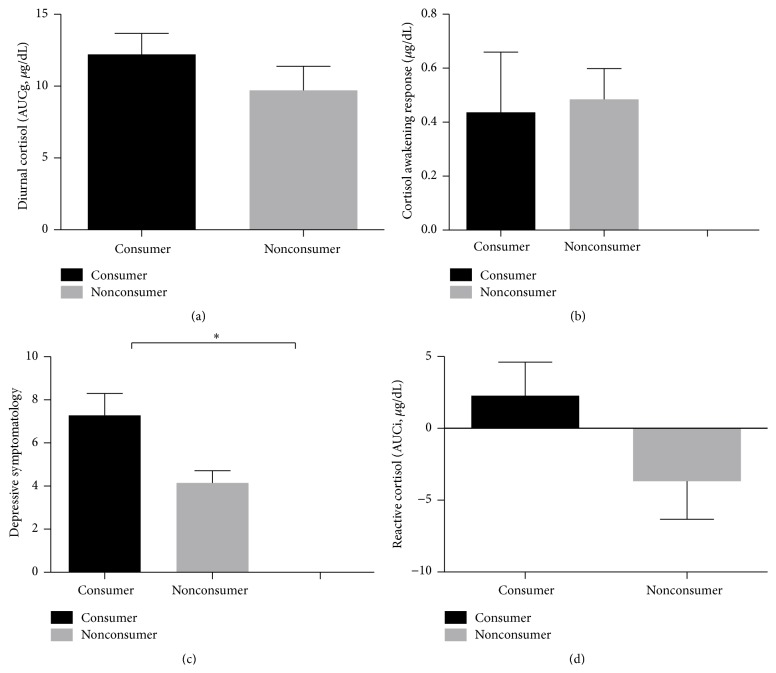
(a) Diurnal salivary cortisol levels (AUCg) as a function of consumer group. (b) Cortisol awakening response as a function of group. (c) Reactive salivary cortisol levels (AUCi) in response to the Trier Social Stress Test as a function of group. (d) Depressive symptomatology as a function of group. The asterisk (*∗*) means *P* < 0.05. For all figures, the error bars represent the standard error of the mean adjusted for sex and body mass index.

We first contrasted consumers and nonconsumers on basal/reactive cortisol levels and depressive symptomatology. We found no differences between consumers and nonconsumers on diurnal cortisol levels AUCg (*F*(1,30) = 0.080, *P* = 0.780; see Figure 2(a)), CAR (*F*(1,30) = 0,31, *P* = 0.862; see Figure 2(b)), and reactive cortisol AUCi (*F*(1,30) = 2.172, *P* = 0.151; see Figure 2(c)). For depressive symptomatology, the analysis showed a significant between-group effect (*F*(1,31) = 6,186, *P* = 0.019), with the consumer group displaying a higher depressive mean score (7,28 ± 1, 01* versus *4,14 ± 0, 57) when compared to nonconsumers (see Figure 2(d)).

### 3.4. Preference for Growth-Oriented or Problem-Focused Books

**Figure 3:**
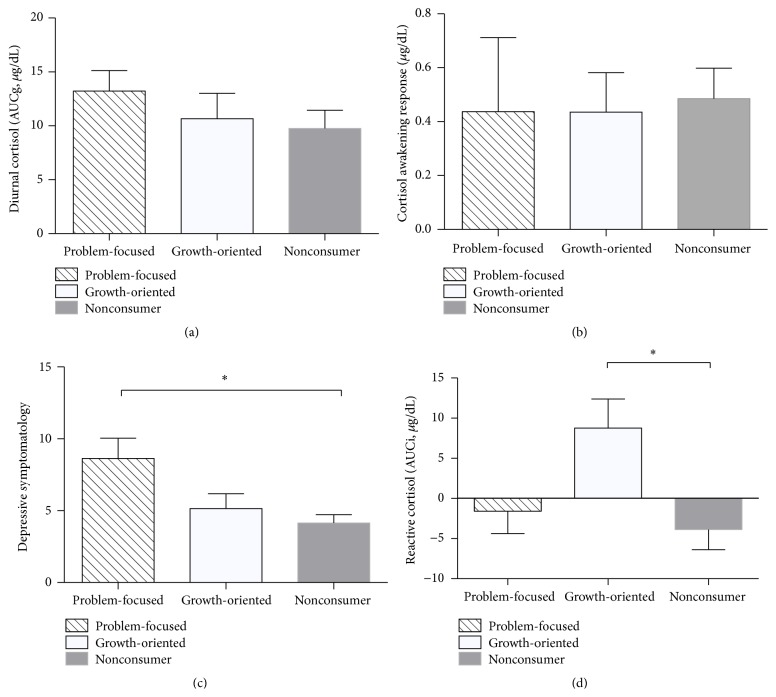
(a) Diurnal salivary cortisol levels (AUCg) as a function of group. (b) Cortisol awakening response as a function of group. (c) Reactive salivary cortisol levels (AUCi) in response to the Trier Social Stress Test as a function of group. (d) Depressive symptomatology as a function of group. The asterisk (*∗*) means *P* < 0.05. For each figure, the error bars represent the standard error of the mean adjusted for age and body mass index.

In a second set of analyses splitting the consumer group into those individuals with a preference for growth-oriented or problem-focused books, we found no group differences in AUCg diurnal cortisol levels (*F*(2,29) = 0.789, *P* = 0.464; see Figure 3(a)) or CAR (*F*(2,29) = 0.015, *P* = 0.985; see Figure 3(b)). We did, however, find a significant group difference in reactive cortisol levels AUCi [*F*(2,29) = 4.079, *P* = 0.028].* Post hoc* analyses showed that the growth-oriented group presented a significantly greater AUCi when compared to the nonconsumer group (*P* = 0.040; see Figure 3(c)). No differences were found between the problem-focused group and nonconsumer group (*P* = 1.00) or between the problem-focused group and the growth-oriented group (*P* = 0.10).

### 3.5. Supplementary Analyses

Strikingly, when one looks at cortisol levels in response to the TSST in the group of nonconsumers (see Figures 1 and 3(d)), one can see that the cortisol response appears to be quite low compared to that of consumers of self-help books. This could represent either a hyporesponse to the TSST in the nonconsumers of self-help books, or a hyperresponse to the TSST in the consumers of growth-oriented self-help books (see Figure 1).

**Figure 4:**
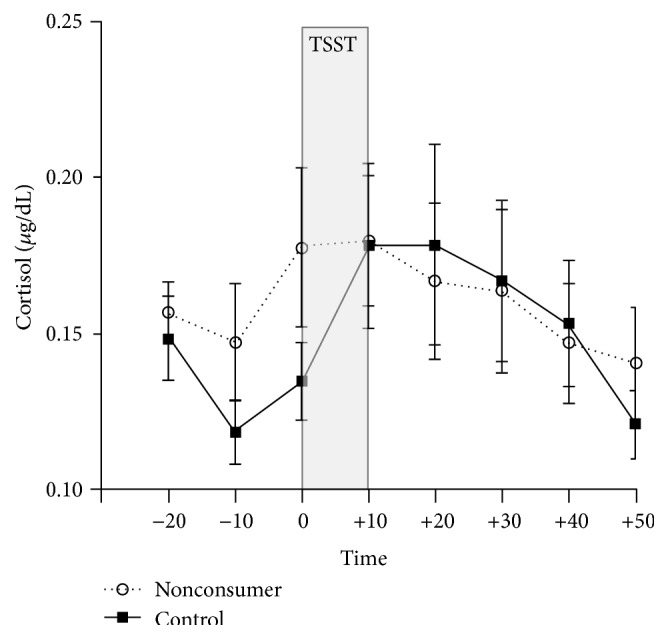
Comparison of reactive salivary cortisol levels in response to the Trier Social Stress Test in nonconsumers of self-help books and a control group of 14 age- and sex-matched individuals extracted from our database. The error bars represent the standard error of the mean.

In order to contextualize the cortisol response to the TSST in the group of nonconsumers, we extracted compiled databases on reactive cortisol in response to TSST (we have more than a thousand participants tested with the same protocol on the TSST in our databases). We extracted data for sex- and age-matched controls and compared their response to the TSST to that of the nonconsumers. The results are presented in Figure 4. We found no significant differences between the cortisol levels in response to the TSST among participants from our previous studies when compared to nonconsumers of self-help books. This suggests that the group of nonconsumers presents a typical cortisol response to the TSST but that the effect seems blunted given the hyperreactivity observed in the group of consumers of growth-oriented self-help books.

### 3.6. Depressive Symptomatology

When we compared groups on depressive symptomatology, we found a group difference in depressive scores [*F*(2,29) = 5.876, *P* = 0.008]. Post hoc analyses showed that the problem-focused group presented a significantly higher score on the BDI than the nonconsumer group (*P* = 0.006; see Figure 3(d)). No differences were found between the growth-oriented group and nonconsumer group (*P* = 0.795) or between the problem-focused group and the growth-oriented group (*P* = 0.095).

### 3.7. Calculation of Effect Size

Cohen's *f* ^2^ effect sizes [45] for group differences on depressive symptomatology were large for both the comparison between consumers and nonconsumers of self-help books (*f* ^2^ = 0.454) and between growth-oriented and problem-focused groups when compared to nonconsumers (*f* ^2^ = 0.63). We found a similar large effect size for the group difference on reactive cortisol levels when comparing the growth-oriented and problem-focused groups to the nonconsumer group (*f* ^2^ = 0.507).

Table 1 presents the effect size for all the comparisons performed in the present study. We also calculated the number of participants that would be needed in a future larger scale study in order to have sufficient statistical power to find group differences on the variables tested. This analysis showed that between 150 and 1000 participants would be needed to find any significant differences in basal cortisol levels as a function of self-help book consumption. By contrast, a much smaller sample size would be needed for reactive cortisol levels (*N* = 40) and depressive symptoms (*N* = 30), based on the medium/large effect sizes found in this small pilot study.

## 4. Discussion

The first goal of this pilot study was to determine whether consumers and nonconsumers of self-help books differ in physiological and/or psychological markers of stress. We found no differences in basal and reactive cortisol levels but reported that consumers of self-help books present increased depressive symptomatology when compared to nonconsumers of self-help books. Although this difference was obtained with a small sample size, the effect size of the difference was large (*f* ^2^ = 0.454). This first result confirms previous suggestions stating that individuals may buy self-help books in order to self-diagnose and/or treat their psychological distress.

The second goal of this pilot study was to assess whether the* type* of self-help books one has a preference for is a better marker of physiological and psychological markers of stress than general interest in self-help books as a whole. First, we found that consumers of problem-focused self-help books presented significantly more depressive symptoms than consumers of growth-oriented self-help books. Hereto, the effect size obtained was large (*f* ^2^ = 0.633). This later result shows that the group differences observed between consumers and nonconsumers of self-help books on depressive symptoms is mainly driven by consumers of problem-focused self-help books.

The increased depressive symptoms found in consumers of problem-focused self-help books converge with the literature on depressive symptomatology suggesting that these symptoms are associated with higher self-victimization [46]. Future studies on self-help books consumers should therefore measure self-victimization in order to verify if it mediates the association between preference for problem-focused self-help books and depressive symptomatology. While we cannot ascertain that consumers of this literature chose to read these kinds of books because they show higher depressive symptoms, it is possible that using this literature leads to higher depressive symptomatology. Since our cross-sectional design does not allow us to determine the directionality of the association found, a longitudinal study would be necessary to test this. Given the large effect size obtained for this group difference in depressive symptomatology, sample sizes in the range of 20 to 30 participants per group would provide sufficient statistical power to confirm group differences.

In future studies of these populations, it could be interesting to assess potential cognitive behavioral tendencies that have been linked to depression. For example, rumination [46], guilt [47], mind wandering [48], and worries [39, 49, 50] are behavioral tendencies among individuals with depressive symptomatology that may be more prominent among consumers of problem-focused books. Indeed, these cognitions and/or behaviors have been shown to be linked to both depressive symptomatology and stress physiology and could act as mediators in the association between problem-focused self-help books consumption and presence of higher depressive symptomatology. Measuring them in future studies could therefore strengthen our understanding of the psychoneuroendocrine profile of consumers of problem-focused self-help books.

The groups did not differ on diurnal cortisol levels, when consumers were compared to nonconsumers and when the consumer group was split as a function of preference for problem-focused or growth-oriented self-help books. Also, the effect sizes for these differences were very low and we calculated that sample sizes between 150 and >1000 individuals would be necessary to find any statistical differences in diurnal cortisol levels between groups. It is important to note that diurnal cortisol rhythm has been shown to be very stable in healthy populations and that most differences observed in basal cortisol secretion are observed in clinical populations [34, 51]. Therefore, the fact that we recruited healthy consumers of self-help books and that we excluded participants presenting psychopathologies might explain why we were not able to detect any differences in terms of diurnal cortisol levels. Therefore, in future studies, it would be interesting to compare the diurnal cortisol profile of clinically depressed individuals who consume self-help books and clinically depressed nonconsumers if one is interested in measuring diurnal cortisol levels as a function of consumption of self-help books.

When we compared groups on reactive cortisol levels, we found that consumers of growth-oriented self-help books are significantly more reactive to a laboratory psychosocial stressor when compared to consumers of problem-focused self-help books or nonconsumers of self-help books and the effect size was large for this group difference (*f* = 0.507). This is an important finding as we had previously found no significant difference between consumers and nonconsumers of self-help books on reactive cortisol levels. This result suggests that it is the preference for a particular type of self-help books (here, growth-oriented self-help books) that is associated with increased production of cortisol in response to a psychosocial stressor and not general attraction toward self-help books more generally.

Interestingly, no group differences were found in the questionnaire testing locus of control. This suggests that the increased stress reactivity to the TSST that we observed in consumers of growth-oriented self-help books cannot be explained by one's belief that one has control over the situation, as suggested by this type of self-help books. One mechanism that could explain this higher reactivity might be some other personality trait inherent to people who are attracted by this type of literature. Even though we measured basic personality traits using the NEO-FFI and did not find any differences for five factors measured, it is still possible that some other personality traits that elude measurement with the NEO-FFI could explain the greater cortisol reactivity reported in individual having a preference for growth-oriented self-help books.

On the other hand, we do know that HPA axis reactivity to stressors plays a critical role in providing energy resources to face the environment and is therefore both adaptive and necessary [52]. Therefore, another possible mechanism that could explain the higher stress reactivity observed in individuals having a preference for growth-oriented self-help books is that coping mechanisms taught in this literature allow these consumers to react in a more effective way to their environment as required by the situation. This suggestion goes along with studies performed in depressed patients [53] and normal individuals [54] showing that greater use of escape-avoidance coping (unhealthy coping mechanism) is associated with less cortisol reactivity.

### 4.1. Limitations

The present pilot study is characterized by a number of limitations, including a small sample size, a cross-sectional protocol, and an underrepresentation of men. Although we made sure that our groups were equivalent in a number of factors that are known to have effects on the physiological stress response (such as sex, sex hormones, socioeconomic status, age, and BMI), it is still possible that some of the negative findings reported here are due to a Type II error due to small sample size. Additionally, while the current pilot study relied on the use of a “daily sampling questionnaire” in order to assess participant's compliance when collecting diurnal cortisol saliva samples, this method has been shown to be less reliable than the use of electronic devices [37]. However, a recent study suggests that multiday sampling somewhat tempers this effect in comparison to only one day of sampling [42, 55]. Future studies on consumers of self-help books should consider using electronic devices in the assessment of diurnal cortisol as this method was shown to be more reliable [29].

Furthermore, even though locus of control did not explain the intergroup differences in terms of stress reactivity and depressive symptoms, other factors such as coping strategies that have not been measured in the present study may have predictive value for cortisol secretion in consumers of problem-focused* versus* growth-oriented self-help books. Future studies assessing psychological and/or physiological markers in consumers of self-help books should therefore consider measuring coping strategies, which may explain some of the observed associations between variables. Also, as mentioned earlier, the cross-sectional design prevents us from determining any directionality between variables and, consequently, a longitudinal design measuring stress hormones before and after utilization of self-help books could help disentangle the cause-effects relationship of the self-help book industry on physiological and psychological markers of stress. Finally, given the differences in psychological and biological markers of stress observed in consumers of problem-focused* versus* growth-oriented self-help books, it would be important in future studies to determine whether one group of consumers benefits more from a particular type of unguided self-help literature when compared to the other group.

## 5. Conclusion

Although we found no general difference in cortisol levels when comparing consumers and nonconsumers of self-help books, we found that consumers of growth-oriented self-help books are more stress reactive when facing a social evaluative threat, while consumers of problem-focused self-help books show higher depressive symptomatology when compared to nonconsumers of self-help books. Our results therefore suggest that preference for a particular* genre* of self-help book (problem-focused* versus* growth-oriented) may be associated with increased stress and/or mental burden in consumers of self-help books. Every year, the self-help industry generates billions of dollars in the US and Canada making it one of the most lucrative businesses in North America. Clinicians are now using guided bibliotherapy to help patients deal with various life conditions and we know that unguided self-help books differ greatly in terms of quality of valid scientific information provided. It is predicted that the self-help book industry will only grow in future years. Consequently, it is essential to understand the impact of different types of self-help books on individuals' physical and mental health.

## Figures and Tables

**Table 1:** Cohen's *f* ^2^ effect sizes for the comparisons of basal/reactive cortisol and depressive symptoms between consumers and nonconsumers of self-help books and between consumers of problem-focused *versus* growth-oriented self-help books when compared to nonconsumers.

	AUC basal cortisol levels	AUC reactive cortisol levels	Depressive symptoms
Consumers *versus* nonconsumers	Cohen's *f* = 0.0821 *N* > 1000	Cohen's *f* = 0.269 *N* = 112	Cohen's *f* = 0.454 *N* = 42

Problem-focused *versus* growth-oriented *versus *nonconsumers	Cohen's *f* = 0.2418 *N* = 168	Cohen's *f* = 0.507 *N* = 40	Cohen's *f* = 0.633 *N* = 30

Cohen's *f* ^2^ represents one of several effect size measures that is generally used in the context of a *F*-test for ANOVA. Cohen gives the following guidelines for the psychological and/or social sciences for Cohen's *f* ^2^ values: small effect size = 0.10; medium effect size = 0.25; large effect size = 0.40.
